# Tailoring the web-based ‘Partner in Balance’ intervention to support spouses of persons with frontotemporal dementia

**DOI:** 10.1016/j.invent.2021.100442

**Published:** 2021-08-10

**Authors:** Jeroen Bruinsma, Kirsten Peetoom, Lizzy Boots, Maud Daemen, Frans Verhey, Christian Bakker, Marjolein de Vugt

**Affiliations:** a Department of Psychiatry and Neuropsychology/Alzheimer Centre Limburg, School for Mental Health and Neuroscience, Maastricht University, Maastricht, the Netherlands; b Department of Primary and Community care, Radboud University Medical Centre, Nijmegen, the Netherlands; c Radboudumc Alzheimer Centre, Nijmegen, the Netherlands; d Groenhuysen, Centre for Specialized Geriatric Care, Roosendaal, the Netherlands

**Keywords:** Partner in Balance, Frontotemporal dementia, Web-based support, Caregivers

## Abstract

Frontotemporal dementia (FTD) typically starts before the age of 65 years, and symptoms differ from other dementias (e.g. Alzheimer's dementia). Spouses are often caregiver and experience difficulty coping with the profound changes in personality and behavior accompanying FTD. Most interventions available to these spouses do not match their need for tailored and flexible psychosocial support. Therefore, tailored content for spouses of persons with FTD was recently incorporated in the proven effective and web-based Partner in Balance intervention.

**Methods:**

This feasibility study prospectively evaluated the tailored Partner in Balance content for spouses of persons with FTD. Spouses followed the 8-week intervention, and qualitative and quantitative measures were used to evaluate expectations and barriers prior to participation and aspects of usability, feasibility, and acceptability of content. Additionally, effects were explored regarding caregiver self-efficacy, sense of mastery, stress, depression and anxiety.

**Results:**

Twenty-seven spouses caring for a spouse with FTD at home started the intervention. Eventually, 20 completed the intervention (74.1%). Partner in Balance matched the expectations of participating spouses and helped them to find a better balance between caregiving and personal life, acquire more peace of mind, and facilitated coping with behavioral and communication difficulties. Before participation, time restraints were identified as a potential barrier, but afterwards spouses positively evaluated the flexibility of the web-based approach that allowed them to participate at a convenient time and place. They valued the recognizability of the videos and narrative stories on FTD. Post-intervention, spouses qualitatively felt more confident, more at ease, and strengthened as a caregiver. Quantitatively, levels of self-efficacy, anxiety and depression significantly improved.

**Conclusions:**

Partner in Balance is a usable, feasible, and acceptable intervention for spouses caring for a spouse with FTD at home. Healthcare organizations could consider adopting Partner in Balance in their daily practice to offer flexible and tailored support to spouses.

## Highlights

• Tailored content on frontotemporal dementia was incorporated in Partner in Balance.
• A pre-post design feasibility study evaluated the tailored content.
• The tailored content and web-based design matched the expectations of participants.
• After Partner in Balance participants felt more confident and more at ease.
• Post-intervention, levels of self-efficacy, anxiety and depression improved.

## 1 Introduction

In 70-80% of the persons with frontotemporal dementia (FTD) the symptoms start before the age of 65 years (Knopman and Roberts, 2011; Rabinovici and Miller, 2010). Given the young age of onset, spouses often perform the role of primary caregiver (Bakker et al., 2013). This is challenging for most spouses because they are likely to be employed (Caceres et al., 2016; Nunnemann et al., 2012), and children may still live at home (Kaizik et al., 2017). Another challenging factor is that persons with FTD are often in good physical condition and the symptoms differ from those of other dementias. Compared to persons with Alzheimer's dementia, the short-term memory often remains relatively intact in persons with FTD. Symptoms more often involve deficits in social cognition, disinhibition, and passive behavior (Shnall, 2009; Lindau et al., 2000). The presentation of FTD is diverse and generally three variants are distinguished. The behavioral variant of FTD is the most prevalent and characterized by personality and behavioral changes such as apathy, disinhibition, compulsive behavior, and a lack of social insight (Onyike and Diehl-Schmid, 2013; Bang et al., 2015). Non-Fluent progressive Aphasia is a FTD variant characterized by difficulty with language production and word comprehension. The semantic variant of FTD is accompanied by aphasia and loss of anomia of words, persons, places, and objects (Bang et al., 2015). In all variants, behavioral and emotional symptoms can occur such as apathy, repetitive behavior, and depression (Bang et al., 2015). Coping with these symptoms is challenging for spouses and linked to burden and distress (Caceres et al., 2016; Kaizik et al., 2017). The young age and symptomatic overlap with psychiatric disorders is known to complicate and delay the diagnosis of FTD (van Vliet et al., 2013; Ducharme et al., 2020). Establishing an FTD diagnosis may last up to 6.1 years, compared to 4.4 years in young-onset dementia in general (van Vliet et al., 2013; Draper et al., 2016). The delay in diagnosis is problematic as it impedes the ability of spouses to adapt to the caregiving role because a diagnosis helps with understanding the changes in their spouse with FTD (Bruinsma et al., 2020).

In the phase after obtaining the diagnosis, spouses of persons with FTD often feel socially and professionally unsupported (Bruinsma et al., 2020; Rosness et al., 2008). For example, they experience that family and friends trivialize the severity of symptoms. Additionally, they often feel that healthcare professionals struggle with providing advice on coping with symptoms of FTD (Bruinsma et al., 2020). The majority of available support for caregivers is designed with the elderly and in particular Alzheimer's dementia in mind, resulting in a mismatch between support and the needs of spouses of persons with FTD (Bruinsma et al., 2021a; Nunnemann et al., 2012). Therefore, spouses postpone the initiation of professional care and support services (Bruinsma et al., 2020). This is problematic because support can facilitate adaptation to the caregiving role. For example, by increasing levels of confidence, and decreasing levels of burden and distress in caregivers (Gossink et al., 2018; Boots et al., 2018). Tailored, accessible, and flexible support services for spouses of persons with FTD may facilitate timely access (Cations et al., 2017). However, due to the low prevalence of FTD it is difficult for spouses to find access to appropriate support close to home. Therefore, web-based support may allow for flexibility and accessibility, also to caregivers living in rural areas (Diehl-Schmid et al., 2013). Previously, web-based approaches already showed potential in improving caregiver well-being (Boots et al., 2014). Therefore, this study used the proven effective web-based Partner in Balance intervention as a starting point. Recent studies showed that Partner in Balance helped caregivers to prepare for the role of informal caregiver. Post-intervention, caregivers improved in self-efficacy, sense of mastery, and quality of life (Boots et al., 2018, Duits et al., 2020, Boots et al., 2016, Bruinsma et al., 2021). The web-based design also showed a good fit for family caregivers of persons with young-onset dementia (Boots et al., 2017). Although these results are promising, spouses of persons with FTD had difficulty recognizing their personal situation in the generic intervention content that primarily addressed spouses of persons with Alzheimer's dementia (Bruinsma et al., 2021). Therefore, tailored content was recently incorporated in Partner in Balance by developing tailored videos, personal stories, and psycho-education for spouses of persons with FTD. This feasibility study evaluates how spouses perceive this tailored intervention content on FTD.

## 2 Methods

This pre-post design feasibility study evaluated tailored content for spouses of persons with FTD that was incorporated in the Partner in Balance intervention. Spouses caring for a spouse with FTD at home participated in the intervention, and qualitative and quantitative measures evaluated expectations and barriers before participation, and perceptions regarding usability, feasibility, and acceptability of the tailored content. Additionally, explorative effects were examined. The CONSORT-EHEALTH was used as a guideline for reporting (Eysenbach et al., 2011).

### 2.1 The Partner in Balance intervention

Partner in Balance is a web-based self-management intervention that aims to facilitate role adaptation by supporting caregivers with finding a balance between caregiving and daily life (Boots et al., 2017; Boots et al., 2018). During the intervention, caregivers receive online coaching from a trained healthcare professional and follow subsequently four self-chosen modules online (Table 1). Caregivers and healthcare professionals access the Partner in Balance intervention via a website, and spent around 6 h on following the intervention in a period of eight weeks. In Partner in Balance there are tailored modules for caregivers of elderly with dementia, young-onset dementia, and Parkinson's disease (Boots et al., 2016; Bruinsma et al., 2021; Duits et al., 2020). Each module includes (1) a video portraying the experiences of other caregivers, (2) narrative stories, psychoeducation, and practical advice, (3) a self-reflection assignment, and (4) a step-by-step change plan to help caregivers to set a personal goal (Boots et al., 2016; Bruinsma et al., 2021).

**Table 1:** Available modules in Partner in Balance.

Modules	Generic modules	Modules on young-onset dementia		Modules on Parkinson's disease	Modules on FTD
Reported in	Boots et al. (2016)	Bruinsma et al., 2021		Duits et al. (2020)	This study
Target population	Spouses	Spouses	Other relatives	Spouses	Spouses
Combining care with work		x	x		x
Impact on family life		x	x		x
Sexuality and intimacy		x			x
Worries about heredity			x		x
Coping with stress*	x			x	
Acceptance	x	x	x	x	x
Balance in activities	x	x	x	x	x
Changes accompanying dementia	x	x	x	x	x
Communication	x	x	x	x	x
Focusing on the positive	x	x	x	x	x
Insecurities and rumination	x	x	x	x	x
Self-understanding	x	x	x	x	x
Social relationships and support	x	x	x	x	x

Note. All modules include a video, narrative stories, psychoeducation, a self-reflection assignment, and a step-by-step change plan to facilitate goal-setting.

**Fig. 1:**
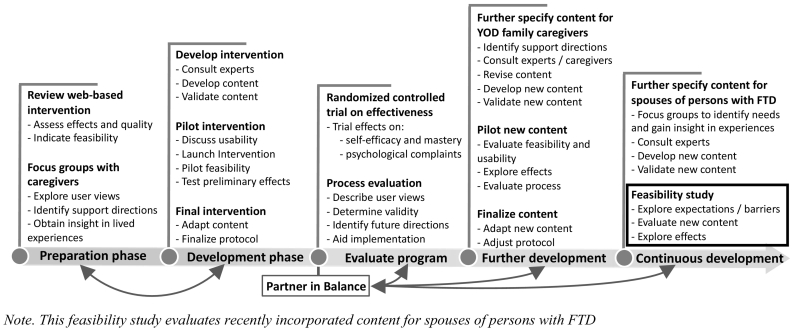
Iterative development process of Partner in Balance. Note. This feasibility study evaluates recently incorporated content for spouses of persons with FTD.

Building upon previous development (Boots et al., 2018; Boots et al., 2017; Boots et al., 2016; Bruinsma et al., 2021), tailored content on FTD was recently incorporated in Partner in Balance. Fig. 1 presents an overview of the iterative development process and incorporation of tailored content on FTD, using the Medical Research Council (MRC) framework (Craig et al., 2008). This feasibility study concerns the final step in the developmental process and evaluated the expectations and perceptions of spouses regarding tailored content on FTD. Tailored content was developed using data from focus group discussions (Bruinsma et al., 2020), and in close collaboration with an advisory committee comprising experts, healthcare professionals, and spouses of persons with FTD. To illustrate, the tailored content consisted of videos and personal stories reflecting the perspective of spouses caring for a person with FTD at home. Additionally, the advisory committee reviewed and supplemented psychoeducation and practical advice. For example, about heredity risks and coping with linguistic or behavioral symptoms of FTD.

### 2.2 Recruitment

Caregiving spouses were eligible for participation in the feasibility study when their spouse had FTD and lived at home. They were recruited via newsletters and social media of the Dutch FTD peer-support organization [*FTD lotgenoten*]. Additionally, dementia casemanagers and psychologists experienced in supporting persons with FTD and their caregivers were recruited to provide coaching during the study. Spouses were asked if their casemanager or psychologist was willing to facilitate coaching. Casemanagers and psychologists were also recruited to coach a spouse from their caseload by spreading information via newsletters, and bi-monthly meetings with healthcare providers affiliated with the Dutch young-onset dementia knowledge centre [*Kenniscentrum Dementie op Jonge Leeftijd*]. Four experienced coaches with a background in psychology from Alzheimer Centre Limburg were also available to coach spouses during the study when needed. Prior to coaching, all casemanagers and psychologists unfamiliar with Partner in Balance received a training comprising an introduction session, eLearning, and a consultation meeting with experienced coaches. On a bi-weekly basis the casemanagers and psychologists were contacted by the research team to monitor progress and verify protocol adherence. This also allowed to pursue a low threshold for support (Christie et al., 2021). Care as usual continued throughout the study.

### 2.3 Measurements

Qualitatively expectations and barriers perceived by spouses before participating in the intervention were explored. Then, quantitative and qualitative measures were used to explore perceptions regarding usability, feasibility and acceptability of the tailored content on FTD. Additionally, it was explored if quantitative effects were in line with those of previous studies on Partner in Balance (Bruinsma et al., 2021; Duits et al., 2020; Boots et al., 2018; Boots et al., 2016).

#### 2.3.1 Participant expectations and barriers before enrolling in the intervention

Pre-intervention, semi-structured interviews of 30 min were conducted to obtain insight in the motives of spouses to participate, their expectations, and potential barriers interfering with participation. To illustrate, questions involved “what persuaded you to participate in Partner in Balance?”, “what are your expectations regarding the intervention?”, and “what might interfere with participation?”. The study was conducted between April 2020 and May 2021, during the Covid-19 pandemic. Therefore, interviews were conducted via telephone and participants were asked about the impact of the pandemic on their role as caregiver, social life, and use of professional support.

#### 2.3.2 Perceptions regarding usability, feasibility and acceptability of the tailored content

Post-intervention, spouses were interviewed for 1 h via telephone using the Program Participation Questionnaire (Boots et al., 2016; Bruinsma et al., 2021). This questionnaire was specifically developed to evaluate usability, feasibility and acceptability aspects of Partner in Balance in the past (Boots et al., 2016). It contains 33 items scored from 1 “strongly disagree” to 7 “strongly agree”. Items covered (1) the use of the intervention in daily life, (2) feasibility of participation, (3) quality of the content provided, (4) experiences with coaching, and (5) perceptions on role adaptation and coping. Throughout the interview, participants were continuously encouraged to elaborate on their experiences. For example, by asking “how did you use the intervention is daily life?”, “what did you like or dislike about the narrative stories?”, and “how can we further improve the intervention materials?”. Additionally, participants were asked about the timing of the intervention and barriers encountered in using Partner in Balance.

#### 2.3.3 Explorative effects

We substantiated our aim to evaluate feasibility by exploring if effects were in line with those of other versions of Partner in Balance (i.e. generic modules, and content on young-onset dementia). Therefore, participants completed a pre-post questionnaire using identical scales as used in previous studies on feasibility, and effectiveness (Boots et al., 2018; Boots et al., 2016; Bruinsma et al., 2021). The pre-post questionnaire explored self-efficacy regarding care-management (six items) and service use (four items) using the Self-Efficacy Scale (CSES). This scale has demonstrated good reliability and internal consistency (Fortinsky et al., 2002). Caregiver mastery was explored with seven items from the Pearlin Mastery Scale (PMS) (Pearlin and Schooler, 1978). A good validity and reliability of the PMS has been demonstrated in diverse populations (Edwards et al., 2000; Marshall and Lang, 1990; Walford-Kraemer and Light, 1984). Ten items measured the amount of stress experienced in the last week by using the Perceived Stress Scale (PSS) (Cohen, 1988). Previously, the PSS demonstarted good internal consistency and validity (Andreou et al., 2011). Anxiety (six items) and depression (seven items) were measured using the Hospital Anxiety and Depression Scale (HADS) (Zigmond and Snaith, 1983). Psychometric properties of the HADS indicate good reliability and validity (Spinhoven et al., 1997).

### 2.4 Analysis

An iterative process was used to analyze the qualitative and quantitative findings from a pragmatic theoretical stance (Morgan, 2014). Therefore, descriptive statistics (means, standard deviation, range) were calculated for items from the Program Participation Questionnaire. To interpret the scores, the interview transcripts were deductively coded by the first author, using Atlas.ti. Codes were summarized in a mind-map and discussed with the second author to derive categories from the data. Then, the findings were discussed with the other authors to substantiate the results. The research team included (neuro)psychologists, health scientists, and a neurologist. All researchers were experienced in conducting qualitative and quantitative research about caregivers of persons with FTD.

To explore if effects were in line with those of previously conducted studies (Bruinsma et al., 2021; Boots et al., 2018; Boots et al., 2016; Duits et al., 2020), the average scores on the pre-post questionnaire were compared for the CSES, PMS, PSS and HADS. This was done in SPSS using paired-sample t-testing to evaluate for statistical significance, using an alpha of 0.05 for two-sided tests (Boots et al., 2016; Bruinsma et al., 2021).

### 2.5 Ethical considerations

The study protocol was approved as a non-medical study by the Medical Ethics Committee of Maastricht University Medical Centre, the Netherlands (METC2019-1286). Prior to participation, all spouses received information about the study, were phoned to see if they had questions, and completed the informed consent procedure.

## 3 Results

### 3.1 Sample

**Fig. 2:**
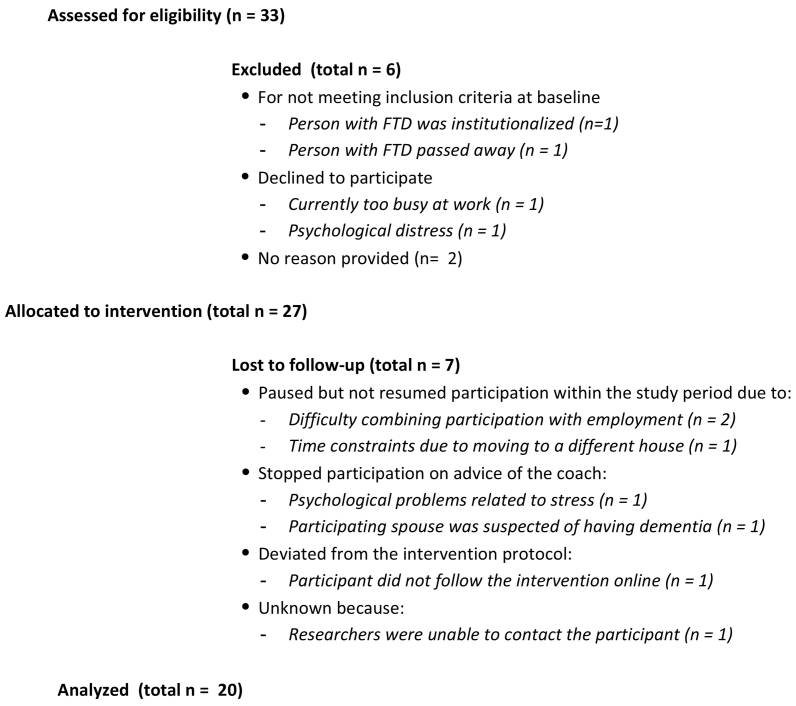
Flow diagram on study participation.

Between April 2020 and May 2021, 33 spouses contacted the research team about participating in Partner in Balance. Twenty-seven out of 33 spouses (81.8%) started the intervention (Fig. 2). Two spouses were excluded prior to participation because their spouse was institutionalized or recently passed away. Four others decided not to participate for various reasons (e.g. currently too busy at work or experiencing psychological distress). Twenty out of 27 participating spouses received coaching from their own casemanager (*n* = 19) or psychologist (*n* = 1). The other seven spouses had a coach without a pre-existing therapeutic relationship from Alzheimer Centre Limburg. The average age of the 27 participating spouses was 64.5 years, ranging from 52 to 81 years. Three of the 27 spouses were male. Eleven spouses were employed, of whom two in fulltime employment. On average, the diagnosis in the person with FTD was established around two years ago, ranging from 3 months to 8 years. Most of the caregiving spouses cared for a spouse with the behavioral variant of FTD (bvFTD; *n* = 15), followed by semantic dementia (SD; *n* = 6), and primary progressive aphasia (PPA; *n* = 4). Two caregiving spouses cared for a spouse with a combination of FTD and amyotrophic lateral sclerosis.

Eventually, 20 of the 27 participating spouses (74.1%) completed the intervention by completing all four self-chosen modules. Spouses who ceased participation attributed this to time constraints or experienced distress. For example, as the result of moving or stress at work. One spouse ceased participation on behalf of advice of her casemanager because she was suspected to have dementia herself.

### 3.2 Expectations and potential barriers

In the interview pre-intervention, most spouses explained they were persuaded to participate by reading about the newly incorporated Partner in Balance modules specifically on FTD. This elicited a sense of recognition regarding themes such as coping with behavioral symptoms, communication difficulties or worries about heredity. Most perceived Partner in Balance as an opportunity to learn more about themselves and acquire more peace of mind by learning more about setting boundaries, coping with challenging behaviors, improving communication skills, and achieving a balance between caregiving and personal life.

“*I want to feel more at ease and achieve peace of mind*. *I hope this* [*Partner in Balance*] *helps me to grow as a caregiver*. […] *I expect the coach to help me to think outside the box*.”

– Spouse of a 61-year-old person a bvFTD diagnosis for 4 months –

Prior to the intervention, most spouses felt confident towards completing Partner in Balance. Some perceived time constraints as a potential barrier, particularly when they combined caregiving with employment. As a result of the Covid-19 pandemic, some spouses worked from home while daycare facilities were closed. In turn, they expected that it would be additionally challenging to find time and privacy to follow Partner in Balance.

“*As a result of the lockdown he is unable to go out and I have to work from home*. *He is very paranoid and constantly wants to know what I am doing*, *who I am calling*. *This is very exhausting*.”

– Spouse of a 54-year-old person with a bvFTD diagnosis for 6 months –

### 3.3 Usability, feasibility, and acceptability

**Fig. 3:**
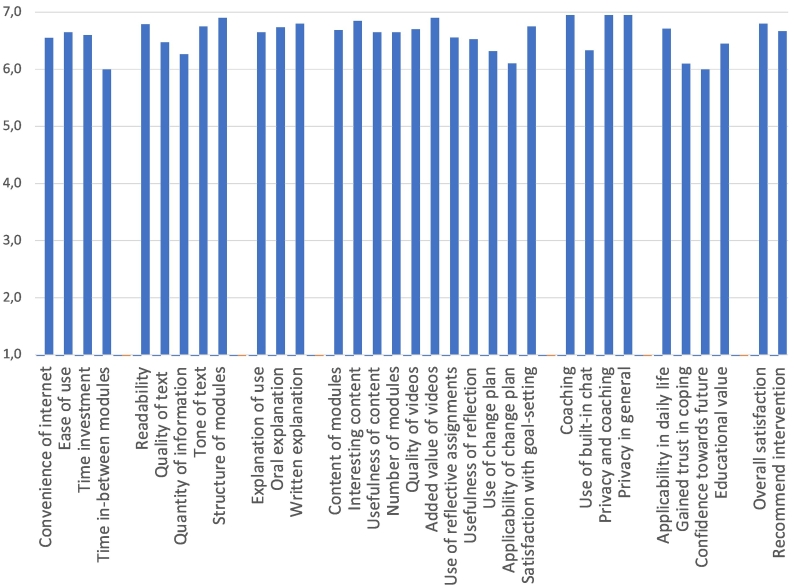
Scoring on the Program Participation Questionnaire.

Post-intervention, all spouses positively evaluated the web-based approach and valued the tailored content on FTD. The sum score on the Program Participation Questionnaire was 217.8, indicating good overall usability, feasibility, and acceptability because the score is higher than the cut-off score of 144. On average, all items were scored 6 or higher, on a scale from 1 “strongly disagree” to 7 “strongly agree” (Fig. 3).

Spouses particularly valued the flexibility offered by the web-based design, enabling them to participate at a convenient time and place. According to spouses they had spent around one and a half hour per module, and it took them around 8 to 10 weeks to complete four modules. Most participants felt this was adequate, and stressed it was important to schedule time to engage in the intervention.

“*The difficulty is you have to find the time and concentration for self*-*reflection and goal*-*setting*. *This can be difficult after a long day*. *You have to schedule time to embed it in your daily routine*.”

– Spouse of a 71-year-old person with a bvFTD diagnosis for one year –

All spouses perceived Partner in Balance as self-explanatory and well-structured. They valued the recognizability of the videos and textual information and appreciated that real caregivers shared their experiences in the videos. Although they perceived each care situation as different, the videos and narrative stories gave them the feeling they were not alone. Some felt the videos could be further improved by adding experiences of a spouse caring for a person with SD, as the current videos only portray spouses of persons with the bvFTD or PPA.

“*The videos give you the feeling you are not an exception*. *You see others openly talk about FTD*. *This encourages you to share your feelings and ask for support*.”

– Spouse of a 54-year-old person with a SD diagnosis for three years –

The participating spouses felt the self-reflection component helped to translate content of Partner in Balance to the personal context. Some felt this helped them to identify their own needs and helped them to prioritize. The goal-setting component in the change plan was often described as the most important element because it helped to set things in motion and apply Partner in Balance in daily life.

“*Self*-*reflection reveals a part of yourself that is normally subconscious*. *This helps you to clarify things*.”

– Spouse of a 72-year-old person with a SD diagnosis for one year –

“*Making a plan helped me to focus my attention and achieve my goals*. […] *I sometimes I wonder why I didn*'*t do this before*.”

– Spouse of a 65-year-old person with a bvFTD diagnosis for four years –

Spouses coached by a healthcare professional with a pre-existing therapeutic relationship felt their bond had strengthened. They attributed this to feeling more comfortable in addressing issues and felt their casemanager or psychologist had a better understanding of the caregiving situation. Some spouses preferred having a coach without a pre-existing relationship. For example, because they did not have a good relationship with their current healthcare professional, or had a need for independent advice from a different angle. According to the participating spouses, the coach also made them feel heard and helped them to see their caregiver role from a different perspective by reevaluating expectations they had towards themselves. The feedback, questions, and advice from the coach also helped to set specific goals for the future.

“*The added value is that I see things from a different angle*. *I feel more confident*. *More comfortable to ask for help when I am in need*. […] *I feel more at ease and less frustrated about his behavior*.”

– Spouse of a 65-year-old person with a bvFTD diagnosis for six months –

Spouses felt the appropriate time to offer Partner in Balance would be in the phase directly after receiving the diagnosis because they had a high need for information in this phase. Some perceived Partner in Balance as a valuable tool throughout the caregiving trajectory because Partner in Balance boosted their confidence and helped them to come to terms with the role of informal caregiver. In retrospect, spouses reported that Partner in Balance matched their expectations as they felt better equipped as a caregiver following the intervention. They felt more at ease and strengthened in managing difficult behavior or communication difficulties. Although most felt more confident, they still experienced uncertainty towards the future due to the unpredictable nature of FTD.

“*Eventually I will be unable to keep up with the progression of FTD*. *It may be a matter of weeks*, *months*, *or years*. *This makes the future unpredictable*.”

– Spouse of a 57-year-old person with a bvFTD diagnosis for four months –

“*It* [*Partner in Balance*] *gives you the confidence you need because you come to the conclusion you are doing the best you can*.”

– Spouse of a 57-year-old person with a bvFTD diagnosis for four months –

### 3.4 Explorative effects

**Fig. 4:**
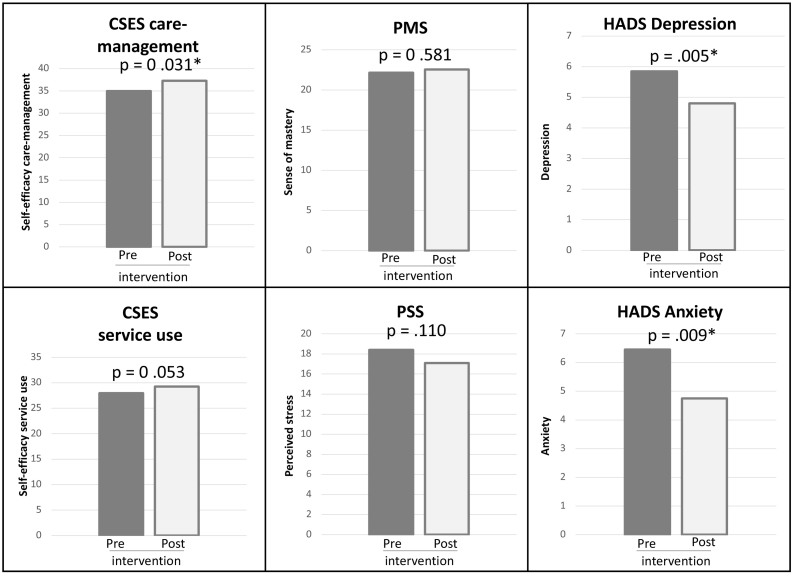
Average scores on the pre-post questionnaire. * = *statistically significant* (*p* ≤ *.05*).

Explorative effects showed that post-intervention the levels of self-efficacy (CSES) regarding care-management (M = 37.3, SD = 8.86) were higher compared to pre-intervention (M = 35.0, SD = 8.96), t(19) = 2.33, *p* = .031. Additionally, post-intervention levels of depression (M = 4.8, SD = 2.28) were lower than pre-intervention (M = 5.9, SD = 2.21), t(19) = -2.926, *p* = .009. Post-intervention levels of anxiety (M = 4.8, SD = 2.67) were also lower than to pre-intervention (M = 6.5, SD = 3.15), t(19) = -3.157, *p* = .005 (Fig. 4).

## 4 Discussion

### 4.1 Key results

The tailored Partner in Balance intervention proved a good fit and matched to the expectations of spouses that cared for a spouse with FTD at home. Caregiving spouses positively evaluated the intervention in terms of usability, feasibility, and acceptability. For example, they valued the use of the intervention in daily life and appreciated the quality of the tailored content provided. Post-intervention, spouses felt more at ease and more confident towards the caregiving role. Explorative quantitative findings showed that spouses improved on self-efficacy, anxiety and depression post-intervention.

### 4.2 Interpretating the findings in the light of previous research

In our study 81.8% of the potential participants started the intervention. This is substantial given that the averaged participation rate in caregiver research is around 27% (Brodaty et al., 2005). In a previous study on Partner in Balance participation rates ranged from 51.9% to 70.2% (Boots et al., 2018). Partly, this may be attributed to the high need of spouses for specific support on FTD (Bruinsma et al., 2020; Rosness et al., 2008). In our study, spouses explained the tailored content on FTD convinced them to participate. They expressed the specific program made them feel recognized. For example, because Partner in Balance gave them the feeling their situation was not an exception. Seeing others talk openly about FTD in the videos also motivated participating spouses to do the same. This is important because spouses of persons with FTD often experience a barrier to discuss FTD with family and friends (Bruinsma et al., 2020). Our findings indicate Partner in Balance helped spouses to mobilize their social network by encouraging them to openly talk about their feelings, and actively ask for support. In turn, this may help to create more understanding in family and friends and reduce the risk of social isolation (Bruinsma et al., 2020). The low availability of other appropriate support may clarify why the tailored content on FTD was evaluated highly positive in our study. The overall score on the Program Participation Questionnaire was 217.8, higher than the cut-off of 144. This quantifies the high level of satisfaction spouses expressed during the interviews because in previous studies on Partner in Balance scores ranged from 195 to 211 (Boots et al., 2016; Bruinsma et al., 2021). The only direction for improvement identified, is the development of new videos portraying spouses caring for a spouse with SD. These new videos will be developed in the near future.

Like previous studies, participating spouses qualitatively confirmed that Partner in Balance made them feel more confident as a caregiver (Bruinsma et al., 2021; Boots et al., 2018; Boots et al., 2016; Duits et al., 2020). The questionnaire quantifies this by showing significant improvement in self-efficacy. Additionally, spouses qualitatively felt more at ease, and quantitatively reported lower levels of anxiety and depression post-intervention. Spouses of persons with FTD are at a high risk for burden, distress, and depression (Mioshi et al., 2013; Diehl-Schmid et al., 2013). This has been attributed to the complexity of coping with emotional and behavioral symptoms that accompany FTD (Kaizik et al., 2017). Spouses may perceive symptoms as uncontrollable and intentional (Polenick et al., 2018). Particularly, because persons with FTD often have a low awareness of disease. According to spouses in our study they felt more aware of the influence of their own behavior, and felt more confident in managing challenging symptoms. This was attributed to self-reflection and goal-setting assignments embedded at the end of each module. To illustrate, participants felt goal-setting facilitated coping by helping them to set boundaries to prevent discussions, respond less agitated, and feel less guilt after an argument. An aim of Partner in Balance is to facilitate role adaptation by challenging caregivers to be resourceful, reevaluate their expectations, and think in terms of possibilities instead of limitations. This may have helped spouses in our study to become more resilient, explaining why spouses felt more confident and more at ease after the intervention. Resilience is known to mitigate feelings of anxiety and depression (Kobiske and Bekhet, 2018). Anxiety and depression in spouses of persons with FTD may also be attributed to anticipatory grief. The changes in personality accompanying FTD often give spouses the feeling they already lost their loved one, resulting in feelings of grief (Kaizik et al., 2017). Potentially, Partner in Balance may facilitate the grieving process by helping spouses to come to terms with their feelings, and the role of informal caregiver. This may explain why they experienced less feelings of anxiety and depression.

Like previous studies, spouses perceived a stronger therapeutic relationship if their healthcare professionals provided coaching (Boots et al., 2017). More specifically, they felt recognized and heard by their coach. They particularly appreciated the constructive feedback, practical advice, and support from the coach. This is important because spouses of persons with FTD often perceive low levels of professional support, undermining their confidence in care and support services (Bruinsma et al., 2020; Johannessen et al., 2017). Currently, only little guidance is available for healthcare professionals aimed at providing psychosocial support to caregivers of persons with FTD (Shnall, 2009). To facilitate coaching in our study, healthcare professionals were recruited via interested spouses, and by spreading information directly to healthcare professionals. Throughout the study, 19 casemanagers were willing to coach. Compared to a previous feasibility study on generic modules on young-onset dementia, the number of casemanagers was higher (Bruinsma et al., 2021). This may reflect a need for tools that healthcare professionals can use in supporting FTD caregivers.

In the Netherlands, dementia casemanagers have a vital role in providing psychosocial support and they facilitate access to information, care and support services. Therefore, casemanagers are crucial in promoting and offering tailored support to caregivers, also to spouses of persons with FTD. A previous process evaluation already demonstrated casemanagers perceive a generic version of Partner in Balance as relevant, usable and feasible in daily practice (Boots et al., 2017). Future implementation should therefore focus on getting healthcare professionals acquainted with Partner and Balance and enable them to work with the intervention on a structural basis. It is important for sustainable implementation to adequately position the intervention within the current healthcare infrastructure. In the Netherlands, healthcare organizations receive a budget per patient and can partly allocate this to caregiver support. Therefore, a business model for sustainable implementation was developed enabling healthcare organizations to license Partner in Balance per caregiver (Christie et al., 2020). The non-profit license is used to cover expenses for website maintenance and technological support. Additionally, healthcare organizations purchase tailored trainings for their healthcare professionals who will serve as Partner in Balance coaches throughout the intervention. The training is adapted to the specific organizational context and includes an introduction session, eLearning, and a consultation meeting with experienced Partner in Balance coaches. For caregivers who are supported by healthcare organizations unable to allocate budget for a license, Alzheimer Centre Limburg endeavors to cover the licensing costs through crowd-funding initiatives when possible. Additionally, there is a free web-based alternative without coaching provided by the RHAPSODY intervention for caregivers of persons with young-onset dementia (Kurz et al., 2016). Recently, a Dutch version of the skill-building RHAPSODY intervention has been launched on the website of the Dutch Alzheimer Association [*Alzheimer Nederland*].

### 4.3 Strengths and limitations

In this study we were able to include a diverse sample in age and level of employment. Additionally, spouses of persons with different variants of FTD participated, namely bvFTD, SD, and PPA. Two spouses caring for a spouse with a combination between FTD and amyotrophic lateral sclerosis were also included. The varied sample allowed obtaining a good impression of how the tailored content meets the various needs of FTD caregivers. We substantiated our aim to explore if effects were in line with those of previous studies on Partner in Balance (Boots et al., 2018; Boots et al., 2016; Bruinsma et al., 2021; Duits et al., 2020). We realize that our findings are not sufficient to provide claims about generalized effects given the small sample with limited statistical power. We believe that combining qualitative and quantitative results and relate them to previous findings provides good support for the potentials of Partner in Balance intervention for this target group. Funding is acquired to further estimate long-term effect and health-economic impact of Partner in Balance by conducting a randomized controlled trial. This study will compare an intervention arm of providing Partner in Balance (i.e. both generic and tailored YOD and FTD content) to a control arm providing usual care.

A strength of our study is that most spouses received coaching from their own casemanager, and care as usual continued. Although this may have impacted our findings, this increases the external validity as it resembles how Partner in Balance is perceived in the context of daily practice. For future studies, it would be interesting to evaluate how effects of Partner in Balance may be enhanced by other care and support services, such as peer-support or daycare. According to participating spouses, these services were only limited available during our study due to the Covid-19 pandemic. This may have biased our findings because limited availability of support may influence experienced levels of self-efficacy, mastery, stress, anxiety and depression. However, our findings also show that web-based support has potential in supporting caregivers during times of the Covid-19 pandemic (Duits et al., 2020).

## 5 Conclusions

Partner in Balance is a usable, feasible and acceptable intervention for spouses of persons with FTD. Spouses qualitatively felt more confident and more at ease following Partner in Balance. Quantitatively they significantly improved on self-efficacy, and experienced lower levels of anxiety and depression. Partner in Balance showed to have substantial benefits for FTD caregivers. Therefore, healthcare organizations could consider adopting Partner in Balance in their daily practice to support spouses of persons with FTD at home, especially as a first step in the support process after diagnosis.

## Funding

This study was funded by the Dutch brain foundation [10.13039/501100008358Hersenstichting], grant number BG-FTD-SBB.

## Declaration of competing interest

None.
